# Case Report: Unedited allogeneic chimeric antigen receptor T cell bridging to conditioning-free hematopoietic stem cell transplantation for a child with refractory Burkitt lymphoma

**DOI:** 10.3389/fimmu.2023.1219872

**Published:** 2023-09-06

**Authors:** Xiaomin Yang, Chengjuan Luo, Juan Qian, Xiaohang Huang, Jian Zhang, Jianmin Wang, Changying Luo, Xia Qin, Benshang Li, Jing Chen

**Affiliations:** ^1^ Department of Hematology/Oncology, Shanghai Children's Medical Center, Shanghai Jiao Tong University School of Medicine, Shanghai, China; ^2^ Department of Pediatric Intensive Care Unit, Shanghai Children's Medical Center, Shanghai Jiao Tong University School of Medicine, Shanghai, China

**Keywords:** Burkitt lymphoma, CAR T-Cell Therapy, allogeneic cells, hematopoietic stem cell transplantation, graft-versus-host disease

## Abstract

**Purpose:**

Burkitt lymphoma (BL) is the most common tumor of non-Hodgkin’s lymphoma (NHL) in children, accounting for about 40% of cases. Although different combined short-course chemotherapies have achieved a good effect, refractory/relapsed BL has a poor prognosis with cure rates less than 30%. Chimeric antigen receptor T cell (CAR-T) therapy has developed rapidly in recent years and achieved excellent results in acute lymphoblastic leukemia (ALL). However, in some cases, there is a failure to produce autologous CAR-T cells because of T-cell dysfunction. In such cases, allogeneic CAR-T therapy has to be considered.

**Methods:**

A 17-year-old boy with stage II BL did not respond to extensive chemotherapy and sequential autologous CAR-T therapy. Lentiviral vectors containing anti-CD20-BB-ζ (20CAR) and anti-CD22-BB-ζ (22CAR) transgenes were used to modify the T cells from an HLA-identical matched unrelated donor. Flow cytometry was used to assess the cytokine analyses and CAR-T cell persistence in peripheral blood, enumerated by qPCR as copies per ug DNA. Informed consent for autologous/allogeneic CAR-T therapy was obtained from the patient and his legal guardian.

**Results:**

Unedited HLA-matched allogeneic CD20 and CD22 CAR-T cells were infused after lymphodepletion chemotherapy with cyclophosphamide and fludarabine. The patient experienced Grade IV cytokine release syndrome (CRS) and went into complete remission (CR) after anti-inflammatory treatment including tocilizumab. Because of persistent pancytopenia and full donor chimerism, the same donor’s conditioning-free peripheral blood stem cells were successfully transplanted 55 days post CAR-T. Neutrophils were engrafted at day +11 and platelets were rebuilt at day +47 without obvious acute graft-versus-host disease (GVHD), but there was mild chronic GVHD in the skin and eyes. Currently, active anti-rejection therapy is still underway.

**Conclusion:**

Unedited HLA-matched allogeneic CAR-T cell therapy could be an innovative, effective, and safe treatment for children with refractory/relapse BL without obvious acute GVHD. Conditioning-free allogeneic hematopoietic stem cell transplantation (HSCT) from the same donor is feasible for a patient with full donor T-cell chimerism after allogeneic CAR-T. It cannot be ignored that close GVHD monitoring is needed post HSCT.

## Highlights

● Unedited HLA-matched allogeneic CAR-T therapy could be an innovative, effective, and safe treatment for children with refractory/relapse BL.
● Conditioning-free allogeneic hematopoietic cell transplantation of the same donor is feasible for a patient with full donor T-cell chimerism after allogeneic CAR-T.

## Introduction

Burkitt lymphoma (BL) is the most common tumor of non-Hodgkin’s lymphoma (NHL) in children and teenagers, accounting for approximately 40% (1). Due to good support with high-intensity chemotherapy regimens combined with rituximab, BL can be a truly curable pediatric cancer and is associated with improved outcomes, with survival rates higher than 90% (2). Nevertheless, the prognosis for pediatric patients with relapsed/refractory (r/r) BL remains poor, with an average overall survival rate of less than 30% (3), so improving the curative effect of therapy for these children should be the focus of future research. In recent years, chimeric antigen receptor T cell (CAR-T) therapy has developed rapidly in pediatric acute lymphoblastic leukemia and achieved excellent efficacy (4, 5). In addition, clinical trials have shown that CAR-T has clear efficacy for B-lineage lymphoma (6), including BL in children (7–9).

However, autologous CAR-T therapy might not be effective in BL patients because of T-cell dysfunction, and the biological characteristics of autologous T cells are also negatively impacted by the previous lines of treatment like chemotherapy (10). In the cases in which the production of a CAR-T cell product from autologous T cells has failed, allogeneic CAR-T can be attempted if there is a good HLA-matched donor.

Here, we report the first refractory BL case successfully treated with allogeneic anti-CD20 and anti-CD22 CAR-T therapy, which resulted in sustained complete remission (CR) and manageable cytokine release syndrome (CRS) and successfully bridged to conditioning-free peripheral blood stem cell transplantation (PBSCT).

## Case presentation

### Previous treatment failure

We describe a 17-year-old boy who presented with progressive left-sided cervical lymphadenopathy. Morphology and immunohistochemistry from a lymph node biopsy were compatible with a low-risk subgroup of phase II Burkitt lymphoma. Following multiple doses of higher-intensity chemotherapy (the first round: DA-EPOCH+A+B+A; the second round: R-COPADM+R-CYVE-MTX), the boy reached partial remission (PR) as shown by PET-CT. However, a residual tumor was still present after chemotherapy. Previously, he had received CAR-T therapy. The patient received four rounds of prior single target autologous CAR-T cell therapy. The first round was CD19 CAR-T therapy (2.26 × 10^6^/kg); the patient experienced Grade I CRS and progressed on day 30. The second round was CD20 CAR-T therapy (2 × 10^6^/kg) combined with four sessions of radiotherapy. The third round was CD22 CAR-T therapy (2.26 × 10^6^/kg). The fourth round was CD19 CAR-T (1 × 10^6^/kg) again. Unfortunately, the patient’s condition deteriorated after the last CAR-T cell infusion, with high fever, lung infections, and severe respiratory insufficiency.

Imaging indicated a soft tissue mass about 63.1 mm × 33.4 mm × 72.3 mm in the left parapharyngeal space that led to compression of the oropharyngeal cavity. Considering the patient retained a CD19-positive immunophenotype, CD19 single autologous CAR-T cells (2.1 × 10^6^/kg) were infused successfully, and on day 0 he developed a repeated high fever which lasted for 5 days. With a rapidly reduced neutrophil count, C-reactive protein (CRP) and other inflammatory factors and cytokines were sharply increased: interleukin-6 (IL-6) and interleukin-10 (IL-10) reached a peak on day 4, CRP on day 5, and then they gradually declined. Grade I CRS was observed. However, nasopharynx computed tomography (CT) imaging assessment suggested a poor response to autologous CAR-T therapy on day 20.

One month later, the patient deteriorated with right neck pain and with painful progressive persistent dysphagia for 1 week, and the mass compressed the nasopharynx and oropharynx, leading to acute respiratory distress syndrome (ARDS). The pathology of mass biopsy demonstrated that the CD19 antigen was negative, but CD22 and CD20 were still positive. Imaging assessment suggested that the original mass in his neck had enlarged to approximately 118 mm × 27.1 mm × 55.9 mm. Recurrence and progression were considered.

### Allogeneic CAR-T therapy bridging to conditioning-free HSCT

**Figure 1:**
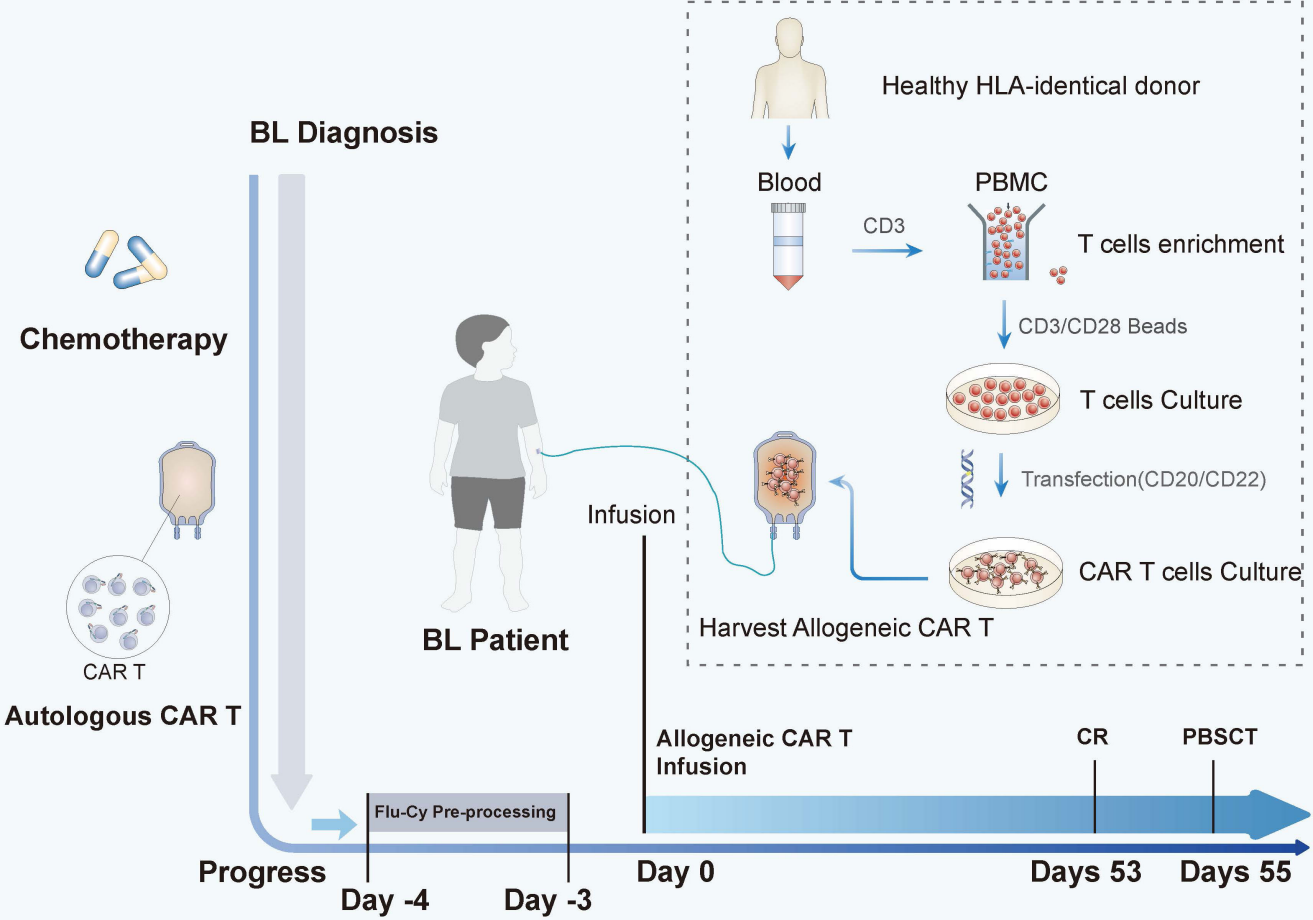
The time line and key events of allogeneic CAR-T bridging HSCT treatment in children with disease progression. After a series of chemotherapy and autologous CAR-T treatments, the disease eventually progressed. On day 0, the patient was infused with allogeneic CD20/CD22 CAR-T cells after conditioning chemotherapy (FLU and CTX were used as pretreatment on day −4 to day −3). The tumor disappeared on day 53. After he had complete remission, he was bridged to allogeneic HSCT on day 55. The allogeneic CAR-T-cell preparation protocol is illustrated inside the dotted lines.

**Figure 2:**
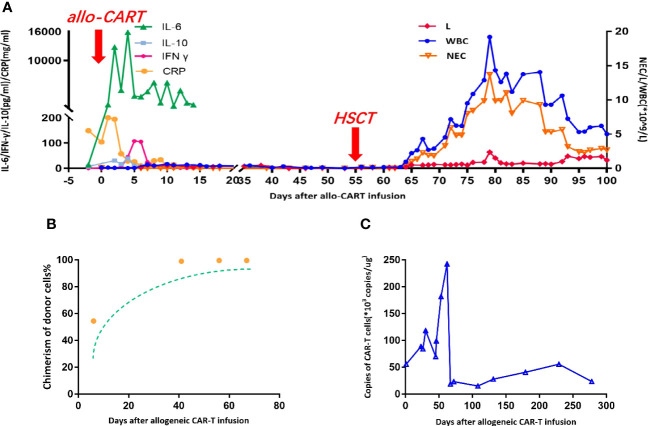
The allogeneic CAR-T treatment outcomes. **(A)** The changing trend of C-reactive protein (CRP), interleukin-6 (IL-6), interleukin-10 (IL-10), and interferon gamma (IFNγ) after allogeneic CAR-T cell therapy. Changes in blood cells before and after HSCT. (*Granulocyte Colony-Stimulating Factor (G-CSF) was infused from day 55 to day 80.). **(B)** Changes in the chimerism of donor cells after allogeneic CAR-T cell therapy. **(C)** Changes in the number of copies of CAR-T cells after allogeneic CAR-T cell therapy. (*The copies of CAR-T cells less than 30*10^3^/ug: CAR-T cells have almost disappeared in the patient.).

We tried to use the patient’s own lymphocytes again to prepare CAR-T, but failed due to poor cell expansion *in vitro*. Chemotherapy (ICE: Ifosfamide + carboplatin + etoposide) combined with radiotherapy was attempted to control tumor progression, but the effect was poor. Our original plan was that the patient would receive HSCT after autologous CAR-T, but he did not get CR after that and we failed to use the patient’s own lymphocytes again to prepare CAR-T. But fortunately, at this time, a fully compatible donor (HLA10/10) supported by the China Marrow Donor Program (CMDP) was available and, after discussion, it was decided to try allogeneic CAR-T therapy. CD20-specific and CD22-specific CAR lentiviral vectors with 4-1BB costimulatory and CD3 zeta signaling domains, respectively, were used to modify the T cells from the donor. On 4 days post allogeneic CAR-T, conditioning chemotherapy (cyclophosphamide 500 mg/m^2^·d; fludarabine 40 mg/m^2^·d) was administered for 2 days to deplete lymphocytes. A month and a half after tumor recurrence, CD20/CD22 allogeneic CAR-T cells were infused with 4.8 × 10^6^/kg (**Figure 1**). The next day, the patient began to have a high fever, combined with hypotension and pulmonary edema, and needed invasive mechanical ventilation, suggesting a severe inflammatory cytokine storm (a grade IV CRS) after CAR-T. The IL-6 level reached 12,780 pg/ml on day 1 (**Figure 2A**), and gradually stabilized after 14 days of treatment with active anti-inflammatory drugs such as tocilizumab and dexamethasone. However, due to continuous pancytopenia, there was still no sign of hematopoiesis recovery more than a month after CAR-T (**Figure 2A**). After allogeneic CAR-T infusion, the patient had been in a persistent state of bone marrow failure and experienced recurrent infections during this time. Several days after CAR-T infusion, the patient experienced pneumonia, air wound infection, and perianal infection. The first bloodstream infection with *Enterobacter cloacae* happened on day 25, and the second bloodstream infection with *Escherichia coli* happened 1 week before HSCT. After the infection was controlled, allogeneic HSCT was undertaken.

Although with severe pancytopenia, a short tandem repeat (STR) report indicated that 99.16% of the T cells were from the donor (**Figure 2B**); peripheral blood stem cell transplantation was performed from the same donor without any conditioning. The donor was mobilized by infusing G-CSF for 5 days before transplantation, and the quantity of the graft was 205ml (about 10grams) in total. Cyclosporine (CsA) and mycophenolate mofetil (MMF) were used for GVHD prophylaxis. Neutrophils gradually recovered as scheduled 11 days after transplantation, and platelets at day +47, and the tumor disappeared at day +53. During the course of the disease, bloodstream infections, such as *Stenotrophomonas maltophil* and *Enterobacter cloacae*, and local infections of the trachea incision, such as *Klebsiella pneumoniae* and *Candida tropicalis*, occurred and active antibiotic treatment was not effective. Therefore, immunosuppressive therapy was tapered off from day +20 to +56 (MMF was from day 0 to day 20, CsA was from day 10 to day 56) due to the serious infection.

After infection was controlled, it was unfortunate that GVHD prophylaxis was not added in a timely manner, and that the child did not receive outpatient follow-up until he was readmitted for infection 8 months after transplantation. Anti-rejection therapy with CsA and hydrocortisone was started on day +342 for 1 month. Combined immunosuppressive therapy (MMF + ruxotinib) was used all the time after that. At present, nearly 2.5 years after transplantation, the primary tumor is still in remission. But mild chronic GVHD in the skin and eyes have occurred successively since 1 year after transplantation, and regular anti-GVHD treatment is still needed.

## Discussion

Autologous CAR-T cell therapy can persist for a longer time and have better tumor-killing activity compared with allogeneic CAR-T because of the absence of the allogeneic reaction (11). Autologous CAR-T therapy has been extensively used in the treatment of hematological cancers, especially in B-lymphoid tumors (12). However, the successful manufacturing of CAR-T depends on the activity of autoimmune cells. Patients who repeatedly receive high-intense chemotherapy always fail to generate CAR-T cells (10). The ability to use cells from healthy donors, referred to as allogeneic CAR-T cells, could potentially address these issues. Although the difficulty of CAR-T cell preparation can be reduced, allogeneic approaches are associated with two major issues. First, the administered allogeneic T cells may cause life-threatening GVHD (13). Second, these allogeneic T cells may be rapidly eliminated by the host immune system, limiting their antitumor activity. Some strategies have been reported for administering allogeneic CAR-T cells, such as the use of virus-specific memory T cells, non-αβ T cells, gene editing with TCR deletion in αβ T cells, donor-derived allogeneic T cells in stem cell transplant recipients, or induced pluripotent stem cells (iPSCs) as a source of allogeneic CAR-T cells (14, 15).

Based on clinical experience, the ability of allogeneic CAR-T cells to eliminate tumor cells is dependent upon the initial expansion, duration of persistence, absence of GVHD, and the ability of the host immune system to reject these cells. It is possible to use T cells without processing as allogeneic CAR-T therapy if there have been well-matched donors. Therefore, we found a suitable donor and tried allogeneic CAR-T cell therapy. The child was in a state of severe immunodepression after several intense immunosuppressive treatments, which made the allogeneic CAR-T continue to be active in his body for more than 2 months (**Figure 2C**), and he achieved CR without acute GVHD. These results indicate that CAR-T cells derived from unedited lymphocytes from well-matched donors could be safe and have a long-term and effective killing effect on BL.

Due to the high tumor burden before CAR-T, the child developed severe CRS (Grade IV) with increased cytokines, persistent high fever, and decreased blood pressure. Although the tumor was gradually cleared after allogeneic CAR-T therapy, there were some side effects that may have been caused by CRS, such as pancytopenia and transfusion dependence. The result was consistent with our own experience and literature reports. Compared with leukemia patients, lymphoma patients with severe CRS after CAR-T tend to have longer pancytopenia and a more difficult recovery. Kai Rejeski (16) reported 12 cases of lymphoma in Europe with bone marrow failure after CAR-T who received autologous or allogeneic CD34 cell infusion, and granulocyte reconstitution was achieved in 92% of the patients, platelet reconstitution in 70%, and long-term survival was achieved in nearly half. Recently, the same author also put forward the concept of Immune Effector Cell Associated Hemato-Toxicity (ICAHT) following CAR-T therapy. The management of ICAHT can broadly be separated into an initial phase which aims to mitigate the risk of infections and/other complications, as well as a later phase that is initiated in case of persistent and/or therapy-refractory cytopenias (17). The possibility cannot be completely ruled out that bone marrow failure is caused by GVHD because of the T cell engraftment. For the severe bone marrow failure, the child received HSCT from the same donor 55 days after allogeneic CAR-T. Unlike traditional transplantation, the patient had severe bone marrow failure before transplantation, and nearly all the T cells in the patient after allogeneic CAR-T were from the donor, so we directly infused the donor cells without any conditioning. The neutrophil recovered 2 weeks after transplantation. If unedited allogeneic CAR-T therapy is selected, we need to be very vigilant and prepared for possible bone marrow failure. These results suggest that it is necessary to preserve lymphoma patients’ own stem cells or prepare for allogeneic hematopoietic stem cell transplantation before allogeneic CAR-T.

Graft-versus-host disease (GVHD) is a pathological process caused by the recognition and attack of donor T cells on host target organs (18). GVHD is the most common complication after allogeneic hematopoietic stem cell transplantation. With the application of allogeneic CAR-T technology, GVHD produced by allogeneic CAR-T has been a concern. In patients with relapse after allogeneic hematopoietic stem cell transplantation, donor-derived allogeneic CAR-T has been shown to have similar effects as autologous CAR-T in a progressively reestablished donor immune state (19), whereas clinical studies of allogeneic CAR-T from novel donors without *in vitro* editing are rare. In this case, due to severe immunosuppression after multiple rounds of chemotherapy and CAR-T, allogeneic CAR-T cell infusion rapidly expanded *in vivo* and maintained 99.16% of the donor T cells for a long period of time, which provided an immunotolerant environment for the engraftment of donor hematopoietic stem cells without conditioning. It has been reported that the risk of GVHD will be reduced if the host is in a state of high immune deficiency due to long-term seriously low immunity (20). In addition, some researchers have explored the mechanism by which allogeneic CD19 CAR-T cells mediate anti-lymphoma activity without significantly increasing the incidence of GVHD (21), and found that allogeneic CD19 CAR-T cells expressing CD28 costimulatory molecules undergo enhanced stimulation, leading to gradual loss of effector function and proliferation potential. Thus, the occurrence of GVHD was significantly reduced. The survival time of normal allogeneic CAR-T cells *in vivo* is about 1 month. Since CAR-T cells express CAR and TCR receptors, they are more likely to be activated and thus suffer from activation-induced cell death failure and apoptosis earlier, and are not likely to produce GVHD (20). Pan et al. (22) also reported that GVHD after allogeneic CAR-T from new donors was mild and very easy to control, which seemed to suggest the feasibility of allogeneic CAR-T prepared from T cells without editing treatment. Now, our experience with more than 20 haplo-identical donor CAR-T cases also suggests that there is little obvious acute GVHD (aGVHD) post unedited allogeneic CAR-T therapy (data not reported). Unfortunately, when the CAR-T cells in the child’s body were at a very low level (**Figure 2C**) and he did not experience CRS caused by CAR-T cells for a long time, it was most likely due to the early cessation of immunosuppressive therapy to control the severe infection and the lack of timely intervention when GVHD occurred; chronic GVHD developed and the patient to date still needs immunosuppressive therapy (IST) even with long-term complete remission. This suggests that close monitoring of GVHD is important.

## Conclusions

Our patient was the first case with refractory/relapse BL who achieved complete remission after unedited allogeneic CAR-T cell treatment and bridged to condition-free HSCT successfully. We can conclude that HLA-matched allogeneic CAR-T therapy could be an innovative, effective, and safe treatment for those who fail to utilize their own cells. Close follow-up and GVHD monitoring are important.

## Data availability statement

The raw data supporting the conclusions of this article will be made available by the authors, without undue reservation.

## Ethics statement

The studies involving humans were approved by Shanghai children’s medical center. The studies were conducted in accordance with the local legislation and institutional requirements. Written informed consent for participation in this study was provided by the participants’ legal guardians/next of kin. Written informed consent was obtained from the minor(s)’ legal guardian/next of kin for the publication of any potentially identifiable images or data included in this article.

## Author contributions

XY, JQ, and XH performed research and draft the manuscript; CJL, JZ, and JW performed the experiments and participated in the writing of the paper; CYL, XQ analyzed the results; BL, JC designed the research and approved the final manuscript.
